# Experimental Study on the Modified P–V–T Model to Improve Shrinkage Prediction for Injection-Molded Semi-Crystalline Polymer

**DOI:** 10.3390/polym18030349

**Published:** 2026-01-28

**Authors:** Shia-Chung Chen, Yan-Xiang Liang, Chi-Je Ding, Yu-Hung Ting

**Affiliations:** 1 R&D Center for Smart Manufacturing, Chung Yuan Christian University, Taoyuan 32023, Taiwan; shiachun@cycu.edu.tw; 2 R&D Center for Semiconductor Carrier, Chung Yuan Christian University, Taoyuan 32023, Taiwan; 3 Department of Mechanical Engineering, Chung Yuan Christian University, Taoyuan 32023, Taiwan; 4 Moldintel Co., Ltd., Taoyuan 32023, Taiwan

**Keywords:** shrinkage, specific volume, P–V–T model, Tait equation, enthalpy transformation method, solidification time

## Abstract

Shrinkage of injection-molded parts is a major challenge for dimensional accuracy, especially for semi-crystalline polymers where crystallization induces pronounced volume change and heat release during cooling. Because packing pressure is effective only before gate or local solidification, multi-stage packing is commonly used to regulate the overall shrinkage behavior. In practice, however, the solidification/transition temperature taken from standard material tests does not necessarily represent the actual in-cavity state behavior under specific cooling rate and pressure history, which compromises the consistency of P–V–T-based shrinkage prediction. In this study, a modified P–V–T-based framework (Tait equation) is developed for polypropylene (PP) by introducing a Thermal Enthalpy Transformation Method (TETM) to determine a process-relevant solidification time and crystallization-completion temperature (including the corresponding target specific volume) directly from in-cavity melt temperature monitoring using an infrared temperature sensor. The novelty TETM utilizes the crystallization-induced enthalpy release to identify the temperature–time plateau, from which one can identify the effective solidification point. Because the Tait equation adopts a two-domain formulation (molten and solidified states), accurate identification of the domain-switching temperature is critical for reliable shrinkage prediction in practical molding conditions. In the experiment execution, the optimum filling time was defined using the minimum pressure required for melt-filling. Four target specific volumes, three melt temperatures, and two mold temperatures were examined, and a two-stage packing strategy was implemented to achieve comparable shrinkage performance under different target specific volumes. A conventional benchmark based on the solidification temperature reported in the Moldex3D material database was used for comparison only. The results show that the target specific volume determined by the TETM exhibits a more consistent and near-linear relationship with the measured shrinkage rate, demonstrating that the TETM improves the robustness of solidification-time identification and the practical usability of P–V–T information for shrinkage control.

## 1. Introduction

The injection molding process can be divided into five main stages: filling, packing, cooling, mold opening, and ejection. During the injection molding process, polymer melts experience drastic changes in pressure and temperature, which makes the injection molding results unpredictable. The injection molding industry still relies on mold flow software analysis and repeated physical attempts to find suitable production parameters. This approach is not only inefficient, but it also means that the process stability varies depending on the experience of different technicians.

Influenced by Industry 4.0, traditional trial-and-error or empirical manufacturing practices are gradually being replaced by precision manufacturing systems based on scientific principles and real-time process monitoring. These systems enable a higher degree of process control and allow parameter adjustments according to actual molding conditions. To accurately characterize the melt flow behavior inside the mold cavity, key process parameters such as temperature, pressure, and injection speed are essential. Among these, temperature and pressure are two of the most influential parameters in injection molding. It is important to distinguish between directly controlled machine variables and resulting in-mold state variables. Barrel (melt) temperature and mold temperature are directly controlled by the machine settings, whereas cavity pressure and local melt temperature evolve dynamically during the molding cycle as a consequence of flow, viscous dissipation, cooling, and crystallization. Bushk et al. [1] and Zhou et al. [2] reported that the pressure profile during the injection and packing stages strongly influences the final shrinkage of the molded part, and that pressure loss from the gate region is closely related to the degree of shrinkage. Based on this concept, a pressure correction system was developed to compensate for melt temperature deviations encountered in actual mass production. Their results showed that stable production could be restored within five shots using this system, compared with eleven shots without correction, and that the product weight variability was reduced from 0.16% to 0.02%. In the present manuscript, the term “weight accuracy” refers to part-to-part variability under steady-state production conditions after process stabilization, rather than variability during the start-up phase. Zhang [3] used a pressure sensor to study the correspondence between the pressure waveform and the shrinkage deformation in the mold, and found that the mold temperature, melt temperature, and injection pressure were the main factors affecting the pressure in the mold, among which the injection pressure was the most intense, followed by the melt temperature, and finally the mold temperature. Tsai et al. [4] installed pressure sensors at different locations on the runner to confirm the correlation between the flow channel pressure and the pressure in the cavity, so that the sensor could be installed on the runner to observe the stability and quality of the process. The P–V–T properties of polymers have been widely discussed in terms of material characteristics, with the P–V–T curves for amorphous and semi-crystalline materials proposed by Zoller et al. [5] and Berry et al. [6] providing an important foundation for subsequent research. Historically, there were two main techniques for measuring P–V–T, the piston-die technique [7,8] and the confining-fluid technique [5]. The piston-die technique is simpler: it involves placing plastic polymers in a tube, compressing the material inside, and recording the volume before and after compression to determine the material’s compressibility at different pressures. However, due to friction between the pipe wall and the residual material, which affects the accuracy of subsequent measurements [9], the technique has drawbacks. Consequently, the confining-fluid method (also referred to as the limited fluid method in some contexts) was proposed by Quach et al. [10]. This technique involves placing plastic polymers into a closed container, applying pressure, and recording the resulting volume changes. By measuring the pressure-specific volume–temperature (P–V–T) history of the melt in the mold cavity during actual molding, the stability of the process can be confirmed. Johannaber [11] and Wang [12] confirmed that the P–V–T process correlates with the molecular orientation, residual stress, and shrinkage/warpage of the product. Meanwhile, Kamal [13] investigated quality and weight control by measuring gate temperature during solidification and tracking peak mold pressure; both methods were found to effectively stabilize product quality. Similarly, Huang [14] identified that the transition to holding pressure during the isobaric stage significantly impacts quality control. Wu [15] explored the influence of injection conditions on melt shear effects, finding that flow-induced shear alters viscosity, which subsequently affects surface quality and structural strength. In 2011, Wang [16] utilized cavity temperature as a criterion for V/P switching, demonstrating that temperature fluctuations influence product weight and that the duration of the holding stage is critical to final quality. Research by Hopmann et al. [17] in 2016 showed that P–V–T-optimized molding achieves superior surface replication and mechanical performance within the same cycle time. Wang [18] and Bozzelli [19] demonstrated that single-stage packing cannot resolve the issue of varying shrinkage rates across different areas of a product. Furthermore, they showed that multi-stage packing control effectively mitigates these discrepancies, establishing the necessity for multi-stage control. Furthermore, Lu [20] integrated pressure sensors into varying thicknesses of optical product cavities to implement multi-stage holding pressure control, resulting in more uniform product shrinkage. Lou [21] and Chang [22] developed real-time in-mold control technologies, utilizing live temperature and pressure data to generate P–V–T diagrams. Their findings indicated that managing the relationship between mold temperature and holding pressure could improve product quality by 40%. Additionally, Huang [23] applied P–V–T monitoring to crystalline plastics, significantly enhancing their molding quality. Chen et al. [24] proposed a new approach of melt temperature monitoring instead of mold temperature monitoring to predict semi-crystalline P–V–T value in 2023. In addition to that, response surface methodology was utilized to predict the crystallinity–time curve. In their study, P–V–T values of changing processing parameters of melt temperature, packing time, and packing pressure were used to corelate with part shrinkage.

In the field of injection molding, the primary objectives remain improving quality and controllability. While various machine parameters have been extensively studied, the aforementioned literature suggests that in-mold monitoring provides the most significant progress toward these goals. Common quality metrics include weight, warpage, and shrinkage—the latter being the most prevalent. Therefore, this study integrates specific volume values from P–V–T technology with shrinkage rates to evaluate and optimize product quality.

There is a huge difference in characteristic P–V–T response between amorphous [25] and semi-crystalline material around melting temperature range [26]. So, the shrinkage rate is high for semi-crystalline polymer. The shrinkage rate is defined as in Equation (1):

(1) $$\mathrm{Shrinkage}\;\mathrm{rate}=\frac{\mathrm{Lo}-\mathrm{Lm}}{\mathrm{Lo}}\times 100\%$$

where Lo: original length; Lm: measured length.

The modified Tait equations were proposed by Bushko et al. [1] as Equations (1)–(8), where the specific volume is described using a two-domain formulation corresponding to the molten and solidified states.

(2) $$\hat{V}=\hat{V_{0}}\left[1-C\times \ln\left(1+P/B\right)\right]+{\hat{V}}_{t}$$

(3) $$\bar{T}=T-b_{5}$$

(4) $$T_{t}=b_{5}+b_{6}P$$

(5) C=0.0894

(6) $$B=\left\{\begin{array}{c} b_{3m}e^{-b_{4m}\bar{T}},\mathrm{if}\;\;\;\;\;T>T_{t}\\ b_{3s}\;e^{-b_{4s\;}\bar{T}}\;,\mathrm{if}\;\;\;\;\;T\leq T_{t} \end{array}\right.$$

(7) $$\hat{V_{0}}=\left\{\begin{array}{c} b_{1m}+b_{2m}\hat{T}\;,\mathrm{if}\;\;\;\;\;T>T_{t}\\ b_{3s}+b_{2s}\hat{T}\;,\mathrm{if}\;\;\;\;\;T\leq T_{t} \end{array}\right.$$

(8) $${\hat{V}}_{t}=\left\{\begin{array}{c} 0\;\;\;\;\;\;\;\;\;\;\;\;\;\;\;\;\;\;\;,\;\mathrm{if}\;\;\;\;\;T>T_{t}\\ b7\;\;e^{b_{8}\bar{T}-b_{9}P}\;,\;\mathrm{if}\;\;\;\;\;T\leq T_{t} \end{array}\right.$$

where $\hat{V}$: specific volume, $b_{1m}\sim b_{4m}$: resin parameters on melt state, $b_{1s}\sim b_{4s}$: resin parameters on solid state, $b_{5}$: glass transition temperature, $T_{t}$, $b_{6}$: modified pressure coefficient on glass transition temperature, and C: constant 0.0894. The specific volume of the melt can be calculated according to the melt’s instantaneous pressure and temperature during filling, using the modified Tait formula. In this formulation, the transition temperature $T_{t}$, defined as a pressure-dependent function (Equation (3)), determines the switching point between the melt-state and solid-state equations.

While $T_{t}$ can be calculated mathematically from material parameters, in practical injection molding the effective solidification or crystallization-completion temperature does not necessarily coincide with the nominal transition temperature obtained from standard material tests or database values. The actual in-cavity transition behavior is influenced by cooling rate, pressure history, crystallization kinetics, and latent heat release, which may shift the effective domain-switching point of the Tait equation.

In this study, the TETM, described in the next paragraph, is proposed and utilized to combine with a P–V–T monitoring system to investigate the relationship between specific volume and shrinkage rate for semi-crystalline polypropylene (PP). The influence of crystallization behavior on shrinkage is thus captured through the accurate identification of the effective solidification point, improving the applicability of P–V–T-based shrinkage control for practical injection molding processes.

## 2. Materials and Methods

### 2.1. Material

Semi-crystalline Polypropylene (PP), LCY 7533, supplied by LCY Chemical Corp. Co., Ltd., Taipei, Taiwan, with a melt flow index of 5 g/10 min, was used in this study.

### 2.2. Thermal Enthalpy Transformation Method (TETM)

**Figure 1:**
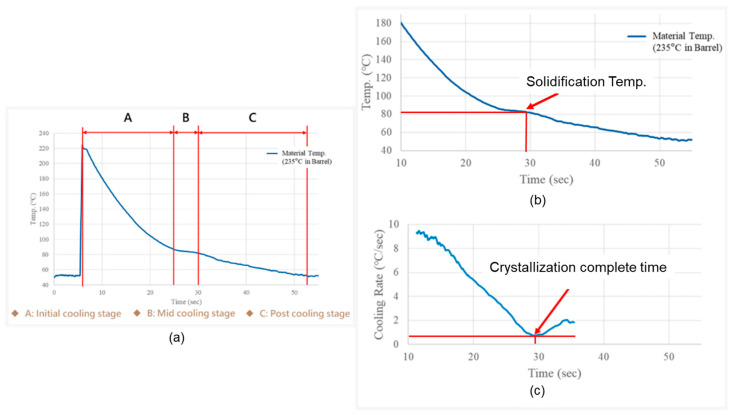
(**a**) Schematic illustration of three stages of melt temperature evolution during crystallization. (**b**) The actual solidification temperature is determined from the temperature plateau region. (**c**) The actual crystallization complete time is determined from the cooling rate profile [24].

The crystallization time and completion temperature are known to be sensitive to part geometry and thermal boundary conditions [21,22,23]. Figure 1 is adapted from the crystallization and enthalpy transition framework reported in Ref. [24] (Figures 4 and 5 in Ref. [24]), which describes the minimum cooling-rate criterion for identifying the end of crystallization. In Stage A, the melt temperature decreases rapidly and the thermal behavior is dominated by cavity cooling. In Stage B, semi-crystalline polymers begin to crystallize and release latent heat, which partially compensates for the heat loss to the mold, resulting in a temporary temperature plateau. In Stage C, after crystallization is completed, the latent heat contribution diminishes and the temperature decreases again with an accelerated cooling rate. In the present study, this framework is simplified and implemented as a practical Thermal Enthalpy Transformation Method (TETM), which identifies the effective solidification point (Stage B–C transition) directly from in-cavity temperature monitoring and is subsequently used to determine the domain-switching point of the two-domain Tait equation. From the slope of the melt temperature variation, one can calculate the cooling rate (Figure 1b). The turning point (the minimum cooling rate) determines the end of crystallization. As a result, the crystallization completion time and the associated transition temperature (solidified temperature) can be known, as shown in Figure 1b,c.

**Figure 2:**
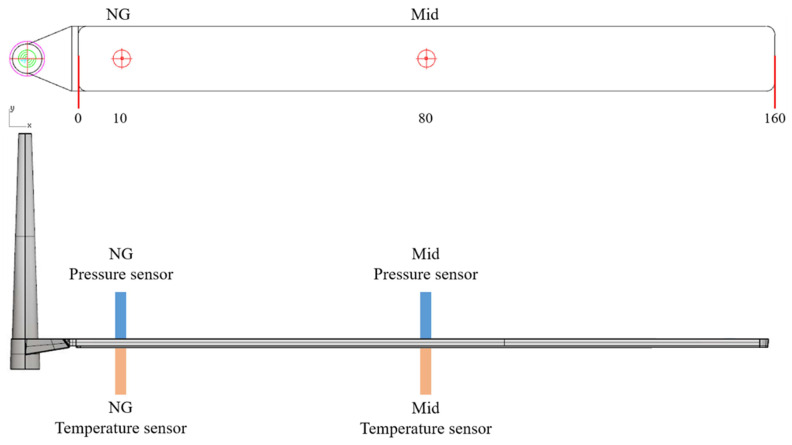
Mold layout to measure the melt temperature and pressure.

**Figure 3:**
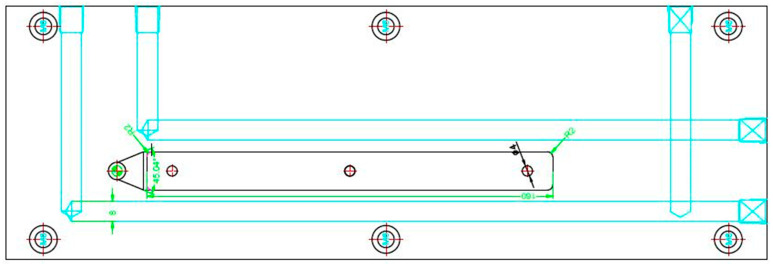
Cooling channel (green color) layout.

**Figure 4:**
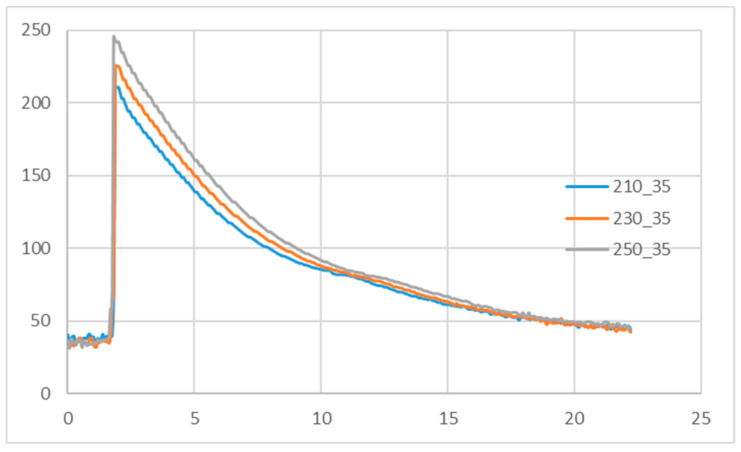
Temperature profiles under different melt temperature at a mold temperature of 35 °C.

**Figure 5:**
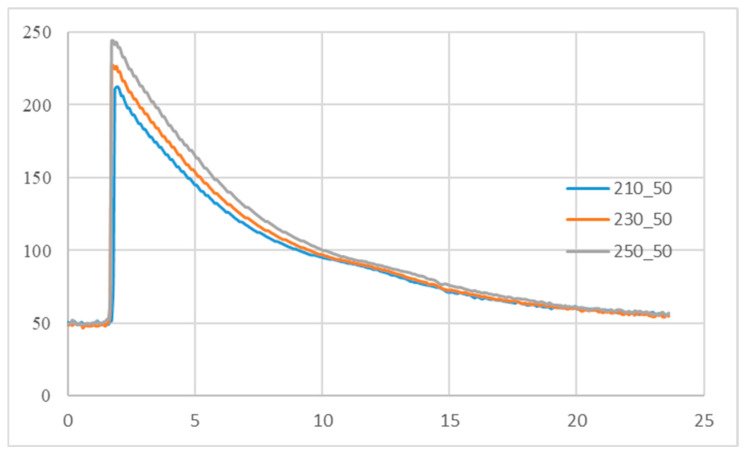
Cooling temperature profile of different melt temperatures at a mold temperature of 50 °C.

The geometry and temperature measurement points employed in this study are illustrated in Figure 2 and Figure 3. Experiments were conducted at three melt temperatures with a mold temperature of 35 °C. The experimentally observed crystallization completion temperatures were 84.1, 84.4, and 84.8 °C, respectively (Figure 4). When the mold temperature was increased to 50 °C, the corresponding completion temperatures were 93.4, 93.8, and 94.9 °C (Figure 5). To ensure comparability among subsequent experiments, the specific volume acquisition temperature was fixed at 90 °C, which lies within the crystallization plateau and represents a process-relevant solidification state under the investigated conditions.

The TETM differs fundamentally from conventional operating window determination. Traditional approaches primarily seek feasible processing conditions that ensure stable molding (e.g., acceptable pressure, temperature, and weight variability), but they do not explicitly define a physically meaningful reference state for extracting specific volume from the P–V–T path. In contrast, the TETM is not intended as a process optimization step; rather, it is a temperature-selection methodology for determining a process-relevant solidification state. Specifically, the TETM leverages the crystallization-induced latent heat release observed in semi-crystalline polymers to identify the temperature–time plateau during cooling. This temperature plateau and the derived cooling rate reflect a material-driven thermal response rather than an arbitrary machine setting. The corresponding specific volume extracted at this state is therefore treated as a target specific volume, which serves as an intermediate variable linking processing conditions to final shrinkage. By grounding the selection of this reference state in the material’s thermodynamic behavior, the TETM provides a more robust and physically interpretable basis for P–V–T-based shrinkage prediction than conventional empirical selection of solidification temperature.

### 2.3. Target Specific Volume

The target specific volume for this study is set for LCY PP-7533. Operating within the acceptable mold and melt temperature range for this crystalline material, a reference group was established using a medium mold temperature of 35 °C and a melt temperature of 230 °C. The injection speed for filling was determined by the lowest point of the U-curve [27]. Cavity pressure and temperature [28] were subsequently acquired using cavity sensors and P–V–T monitoring, allowing for the application of a delayed pressure packing method via the pressure sensor. The solidification time of each section was measured to define the packing time for each stage. Finally, the TETM used the temperature sensor to track the cooling rate, and the measured actual curing temperature of the melt served as the basis for setting the outlet temperature for specific volume extraction. Based on the experimental parameters of the first stage, the actual specific volume of this polymer was measured using P–V–T monitoring technology. This confirmed that the polymer’s minimum specific volume could be achieved within the pressure sensor’s limit. Consequently, the minimum specific volume value was set as the upper limit for the target specific volume, and the actual specific volume from the minimum pressure group was set as the lower limit. These two values defined the four target specific volume values required for the second and third stages.

### 2.4. Injection Molding Machine and Mold

Injection molding was performed using a HSP-100EH2 hybrid (hydraulic-electric) injection molding machine provide by Sodick Co., Ltd., Yokohama, Japan. A long stripe mold with dimensions of 160 × 15 × 3 mm was used to measure the melt temperature and pressure as shown previously in Figure 2 and Figure 3.

### 2.5. Cavity Temperature and Pressure Monitoring, Data Acquisition

A P–V–T data acquisition processor of ADAM-6017 was supplied by Advantech Co., Ltd., New Taipei, Taiwan, which can process the melt temperature and pressure signals. A cavity pressure sensor 6001A and a signal amplifier 5050A were acquired from PRIAMUS System Technologies GmbH, Schwalbach, Germany. A cavity infrared-type temperature sensor by EPSSZT and voltage amplifier EPT-001S were supplied by Futaba Co., Ltd., Mobara, Chiba, Japan. A traditional temperature sensor can only detect the mold surface temperature; however, this infrared temperature sensor can detect the melt core temperature and its function was verified by previous studies [20,21,22].

### 2.6. Shrinkage Rate

Shrinkage rate measurement was achieved by 2.5D profile measurement VM-2515, Power Assist Instrument Scientific Co., Ltd., Taoyuan City, Taiwan.

## 3. Results and Discussion

P–V–T curves provide a wealth of information during the polymer solidification period. Several reports [2,3,4] showed that molded part quality can be predicted by analyzing the P–V–T history. Recently, industry has emphasized the importance of the real filling pattern within the cavity. One can achieve good quality molded parts by monitoring the pressure and temperature profiles. This investigation was achieved by adopting the TETM to study the semi-crystalline polymer crystallization behavior within the cavity. In conjunction with the P–V–T technique developed in our laboratory, the real polymer melt flow history under different melt pressures and temperatures can be better understood. This experiment involves three sections to monitor the specific volume variations and part shrinkage rate within the cavity.

### 3.1. Target Specific Volume’s Temperature Acquisition

According to the P–V–T theory, the difference in specific volume across different segments of the specimen represents the difference in shrinkage. Once the material segment falls below its solidification temperature, its shrinkage rate is no longer affected by the injection system (e.g., packing pressure). Instead, the shrinkage rate will only gradually slow down to a final, specific value as the temperature drops to room temperature. Therefore, the final shrinkage rate of the specimen can be predicted using the specific volume at this solidification temperature. The primary purpose of this stage is to determine this solidification temperature.

In this study, we will compare the stability of the specific volume at different acquisition temperatures. The two acquisition temperatures for the target specific volume value will be the LCY PP-7533 curing temperature provided by the mold flow analysis software Moldex3D and the curing temperature measured by the TETM. If the extraction temperature is too high, the specimen will still be undergoing rapid temperature and pressure change, meaning the specific volume value will also be changing rapidly. Consequently, a stable specific volume value cannot be captured. Furthermore, because the melt has not yet solidified, it will continue to be affected by the operation of the injection system. Therefore, the specific volume value measured at a high temperature cannot effectively predict the subsequent shrinkage.

**Figure 6:**
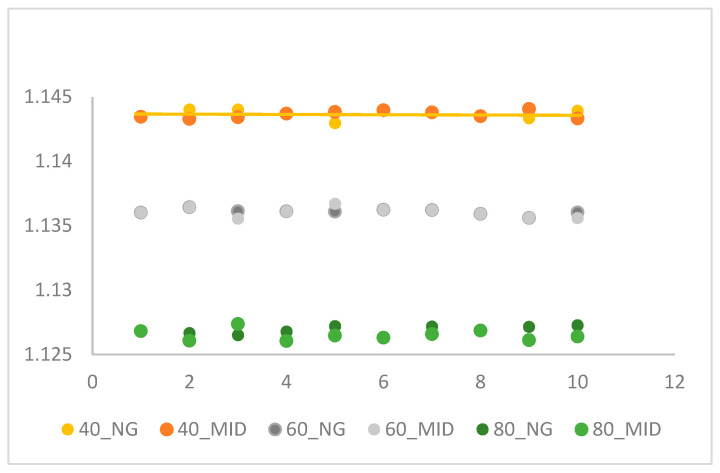
Specific volume distribution measured by the TETM.

**Figure 7:**
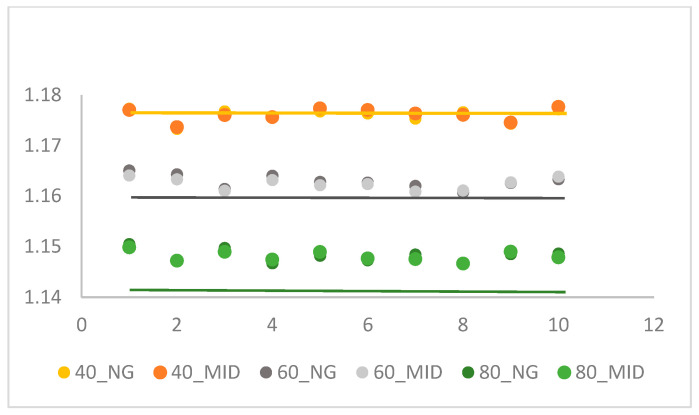
Specific volume distribution by Moldex3D simulation.

**Figure 8:**
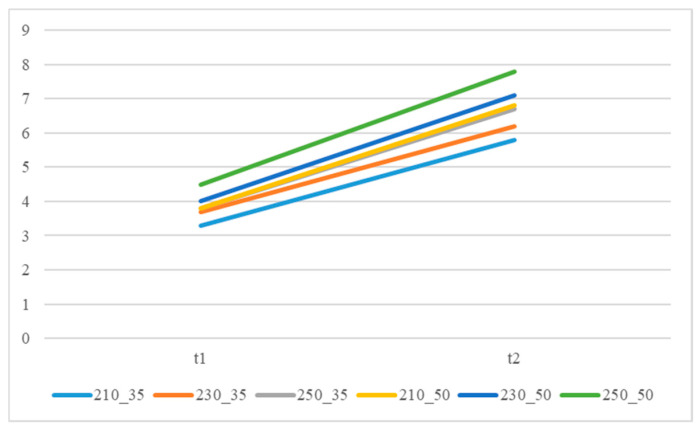
Packing time distribution for different melt and mold temperatures at a thickness of 1.5 mm.

As shown in Table 1, two different acquisition temperatures were used under fixed melt and mold temperature parameters. Three different first packing pressures were tested (40, 60, and 80 MPa), while the second packing pressure was adjusted to align the specific volume between the two stages. Table 2 presents the specific volume stability experiment results from a continuous production of 10 molds using the parameters established in Table 1. Figure 6 and Figure 7 show the specific volume scatter diagrams for the NG (near gate) and MID (middle of specimen) points, respectively, comparing the results obtained using the TETM temperature and the Moldex3D curing temperature across the 10 molds. It is seen in Figure 8 that the specific volume at packing pressure 60 MPA has less deviation between near gate and middle of specimen by the TETM as compared with packing pressure of 40 and 80 MPa, and high packing pressure has low specific volume [24]. The specific volume acquired from Moldex3D software [29] has higher scatter values than those of the TETM. This verified that specific volume by the TETM is more reliable than that of Moldex3D. Based on these results, the temperature derived from the TETM will be used as the extraction temperature for the final outlet specific volume value in subsequent experiments.

### 3.2. Relationship Between Specific Volume and Shrinkage

**Figure 9:**
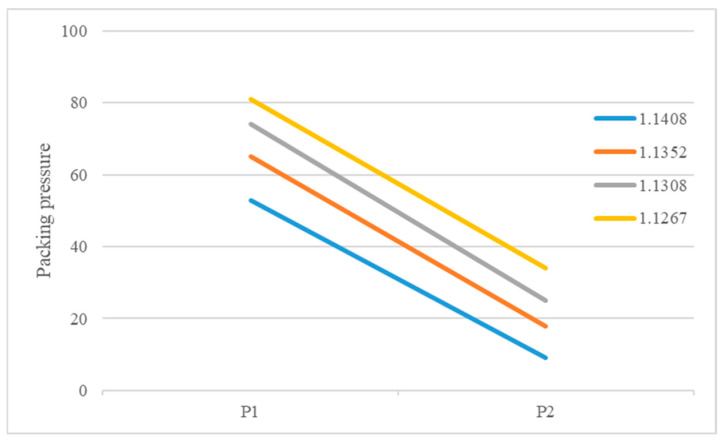
Pressure distribution for different specific volumes at a melt temperature of 230 °C, a mold temperature of 35 °C, and a thickness of 1.5 mm.

**Figure 10:**
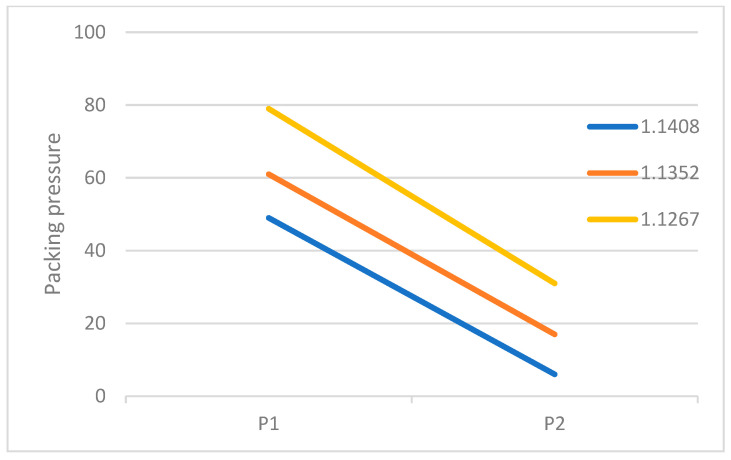
Pressure distribution for different specific volumes at a melt temperature of 250 °C, a mold temperature of 35 °C, and a thickness of 1.5 mm.

**Figure 11:**
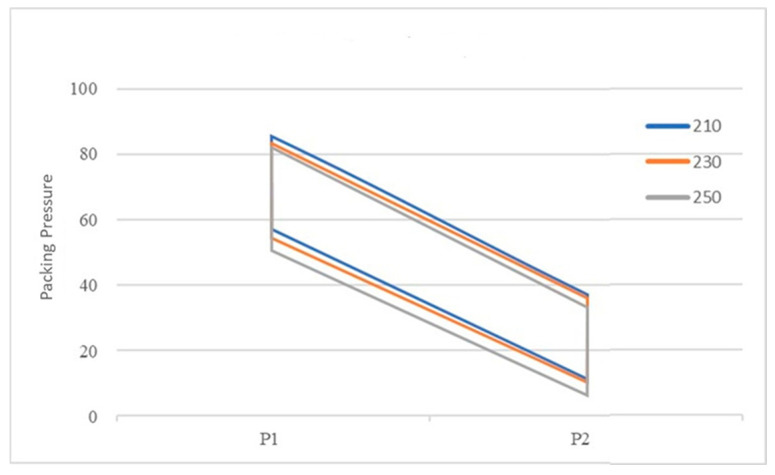
Packing pressure range for different melt temperatures at a thickness of 1.5 mm.

The recommended processing temperature range for the polymers selected in this study is between 190 and 270 °C and the mold temperature range is between 20 and 50 °C. Dosage was defined as the amount injected by continuously increasing the shot size until the sensor-detected pressure rises rapidly, indicating a full shot. Additionally, the lowest point of the U-curve [27] was used as the optimal setting method for the cavity’s filling time. The actual set values for the packing pressure and packing time are shown in Table S1 (in the Supplementary Material). It was found that when using the groups with higher melt and mold temperatures, the required packing time as measured by the delayed pressure method is longer (Figure 8). This is because increasing the polymer’s melt and mold temperatures prolongs the time required for the melt to drop to its crystallization temperature [30], thereby increasing the packing time needed to pack the melt effectively. The packing pressure setting results are summarized in Figure 8, Figure 9 and Figure 10. In all experimental groups, the packing pressure required to achieve a smaller target specific volume exhibits a clear increasing trend [18]. When the results are compared across melt temperatures (Figure 11), groups processed at lower melt temperatures consistently require higher packing pressure to reach the same target specific volume; this trend is observed at both mold temperatures (35 and 50 °C).

**Figure 12:**
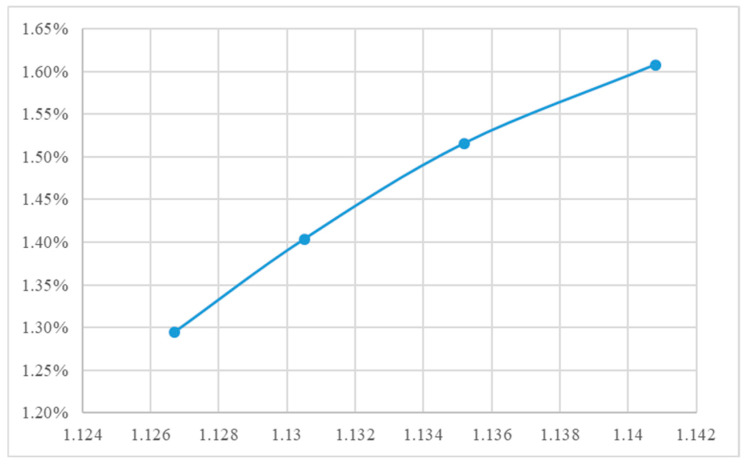
Shrinkage rate vs. four specific volume distributions (thickness 1.5 mm).

Table S2 (see Supplementary Materials) lists the specific volume values obtained under the conditions defined in Table S1. Given the rapid fluctuations of in-cavity pressure and temperature during injection molding and the limitations of data acquisition hardware, minor deviations between measured and target specific volumes were considered acceptable. Table S3 (see Supplementary Materials) presents the segmented shrinkage rates calculated using Equation (1). When the specific volume was controlled using the P–V–T-based approach, the variation in segmental shrinkage was limited to within 0.05%, indicating effective shrinkage regulation across different locations. Figures S1–S4 (see Supplementary Materials) illustrate the shrinkage rates corresponding to four different target specific volumes under varying mold and melt temperatures. By comparing these figures, it is evident that as the specific volume decreases, the shrinkage rate also decreases across different mold and melt temperatures. Finally, Figure 12 demonstrates the linear relationship between these variables; specifically, a higher specific volume correlates with a higher shrinkage rate [31].

### 3.3. Verification of the Relationship Between Specific and Shrinkage Rate

The purpose of this stage is to verify the results of the second stage by maintaining the same boundary conditions but changing the specimen thickness. This aims to verify whether the melt’s flow and cooling behavior at different geometries impacts the specific volume accuracy. For this stage, the specimen thickness was increased from 1.5 to 2 mm, while maintaining the geometric design (a length of 160 mm and a gate-to-specimen thickness ratio of 0.8). All experiments were conducted identically to those in the second stage.

**Figure 13:**
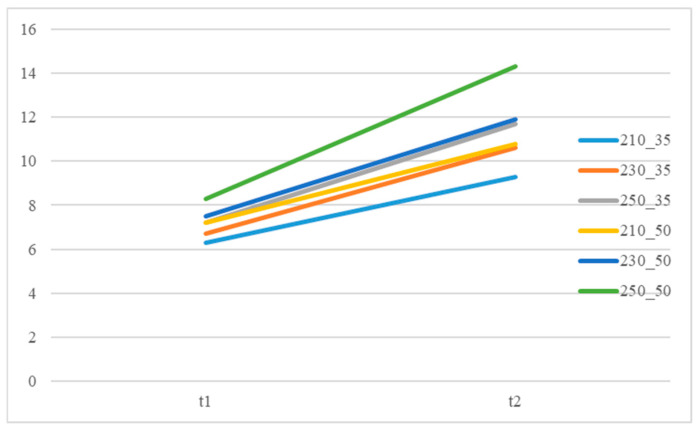
Different melt and mold temperatures vs. packing time profile (thickness 2 mm).

**Figure 14:**
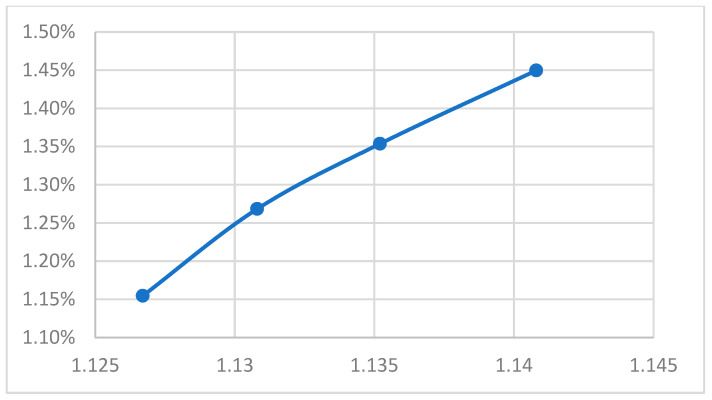
Shrinkage rate vs. four different target specific volumes (thickness 2 mm).

**Figure 15:**
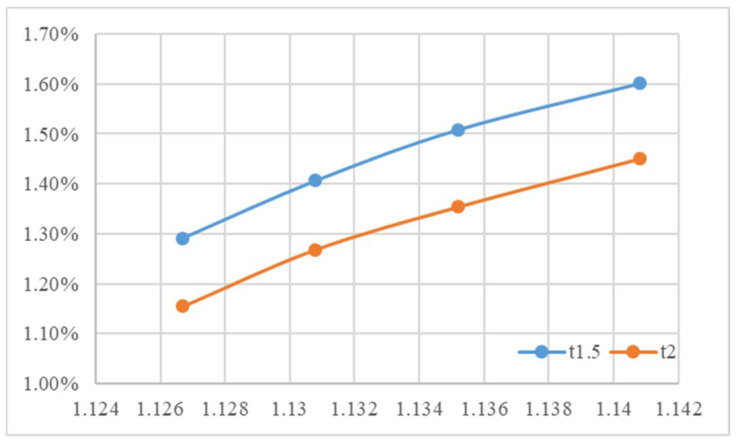
Shrinkage rate vs. four target specific volumes at two different thicknesses.

The actual packing settings used in the final stage are provided in Table S5 (see Supplementary Materials). For the 1.5 mm thick specimens, the required packing time in this stage was longer than in the second stage (Figure 13), reflecting the higher thermal mass and longer cooling path in thicker sections. Although the target specific volume remained unchanged from the second stage, a lower packing pressure was required to achieve it because of reduced flow resistance in the thicker cavity (Figures S5–S7; see Supplementary Materials). Consistent with earlier observations, the low melt temperature condition still demanded the highest packing pressure (Figure S8; see Supplementary Materials). The trend observed in Figure 14, when overlapping these three figures, is consistent with that of the 1.5 mm specimen. This indicates that the required packing time and pressure trends remain similar across different mold and melt temperature settings. Figure 15 illustrates the shrinkage rate versus four target specific volumes at two different thicknesses. These trends are analogous; however, the thicker sample exhibits a lower shrinkage rate, and this trend agrees with the behavior of the amorphous polymer PP [20].

## 4. Conclusions

This study proposes a methodology that utilizes the Thermal Enthalpy Transformation Method (TETM) to determine a process-relevant solidification state based on the actual material’s crystallization behavior. From the variation of melt temperature, the solidified temperature can be inferred and is then integrated with P–V–T control to regulate the linear shrinkage rate of the molded part. In this framework, the target specific volume obtained from the temperature plateau and the cooling rate were identified by the TETM, which reflects the material’s crystallization state rather than an arbitrary thermal reference. The relationship between this target specific volume and the resulting product shrinkage rate is summarized below:

1. Acquisition temperature control: The target specific volume is measured at a specific acquisition temperature. If the acquisition temperature is set too high, the polymer is still in the melt state, causing the specific volume measurement to fluctuate significantly. This is unstable for control. Conversely, using the curing temperature—determined by the TETM as the acquisition temperature—results in a more stable specific volume measurement, making it a more suitable control point.
2. Relationship between specific volume and shrinkage: With the thermal enthalpy curing temperature used as the acquisition temperature, a direct relationship is observed between the measured specific volume and shrinkage. A higher specific volume value corresponds to a higher product shrinkage rate, and conversely, a lower specific volume value leads to a lower shrinkage rate.
3. Target specific volume and product quality: The multi-stage pressure packing capability of the machine allows for the sectional control of the product’s specific volume. By regulating the specific volume of each section to match the target specific volume value, the resulting shrinkage rate across the entire product can be made similar and uniform. Experimental verification, performed across the second and third stages of the study, demonstrated that controlling the specific volume of each part of the product to the target specific volume is equivalent to effectively controlling the final shrinkage rate of the entire product. This confirms the viability of the target specific volume as the primary process control parameter for dimensional stability.

## Figures and Tables

**Table 1:** Experiment setup for different packing pressure by the TETM and simulation.

	Melt Temp. (°C)	Mold Temp. (°C)	Acquisition Temp. (°C)	1st Packing P. (MPa)
A1	230	35	TETM	40
A2				60
A3				80
A4			Moldex3D solidification	40
A5				60
A6				80

**Table 2:** Error of different temperature acquisitions.

	Ave. S. V.		STD S. V.		S. V. Ranges	
	NG	MID	NG	MID	NG	MID
A1	1.1438	1.1437	0.0002	0.0002	0.0009	0.0006
A2	1.1361	1.1362	0.0002	0.0002	0.0006	0.0007
A3	1.1270	1.1265	0.0002	0.0003	0.0007	0.0011
A4	1.1760	1.1762	0.0013	0.0012	0.0040	0.0042
A5	1.1626	1.1628	0.0011	0.0014	0.0031	0.0043
A6	1.1483	1.1482	0.0012	0.0009	0.0038	0.0031

## Data Availability

The raw data supporting the conclusions of this article will be made available by the authors on request.

## Supplementary Materials

The following supporting information can be downloaded at https://www.mdpi.com/article/10.3390/polym18030349/s1, Figure S1: Shrinkage rate profile for different melt and mold temperatures at a specific volume of 1.1408 (thickness 1.5 mm); Figure S2: Shrinkage rate profile for different melt and mold temperatures at a specific volume of 1.1352 (thickness 1.5 mm); Figure S3: Shrinkage rate profile at different melt and mold temperatures at a specific volume of 1.1308 (thickness 1.5 mm); Figure S4: Shrinkage rate profile at different melt and mold temperatures at a specific volume of 1.1267 (thickness 1.5 mm); Figure S5: Pressure profile at a melt temperature of 210 °C and a mold temperature of 35 °C (thickness 2 mm); Figure S6: Pressure profile at a melt temperature of 230 °C and a mold temperature of 35 °C (thickness 2 mm); Figure S7: Packing pressure profile at a melt temperature of 250 °C and a mold temperature of 35 °C (thickness 2 mm); Figure S8: Packing pressure profile at melt temperatures of 210, 230, and 250 °C (thickness 2 mm); Figure S9: Schematic comparison of temperature measurement methods using a Futaba infrared temperature sensor and a conventional thermocouple; Table S1: Packing pressure and packing time setting at a thickness of 1.5 mm; Table S2: Specific volume at a thickness of 1.5 mm; Table S3: Shrinkage rate at a thickness of 1.5 mm; Table S4: Corresponding shrinkage rate at different target specific volumes; Table S5: Packing pressure and packing time setting at a thickness of 2 mm; Table S6: Specific volume at a thickness of 2 mm; Table S7: Real shrinkage rate at a thickness of 2 mm.
