# Supplementary material for: Experimental Study on the Modified P–V–T Model to Improve Shrinkage Prediction for Injection-Molded Semi-Crystalline Polymer
Source: Polymers (Basel). 2026 Jan 28;18(3):349. doi: 10.3390/polym18030349 (PMC12899455; doi:10.3390/polym18030349)
Supplement: Supplementary file 1 [file polymers-18-00349-s001.zip › polymers-4100570-supplementary.pdf]

Supporting Information for this manuscript

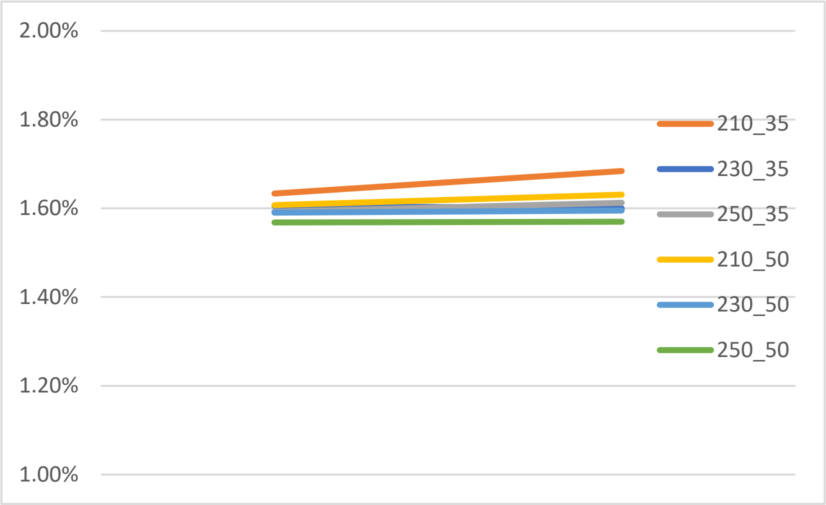

Figure S1. Shrinkage rate profile for different melt and mold temperatures at a specific volume of 1.1408 (thickness 1.5 mm).

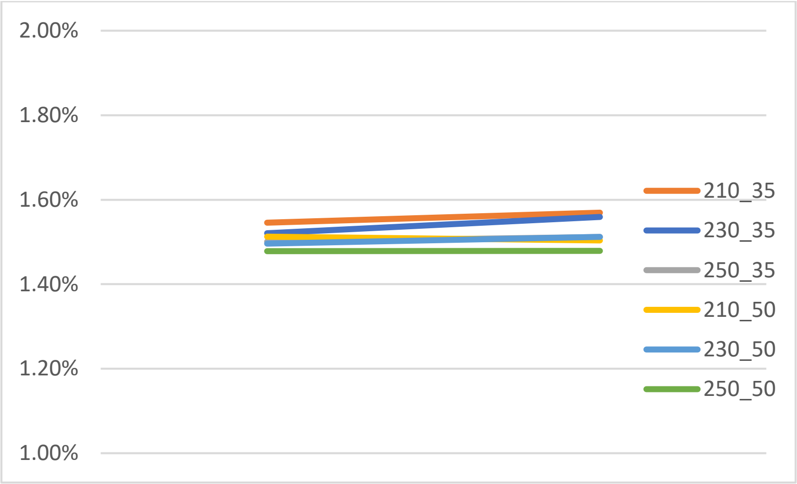

Figure S2. Shrinkage rate profile for different melt and mold temperatures at a specific volume of 1.1352 (thickness 1.5 mm).

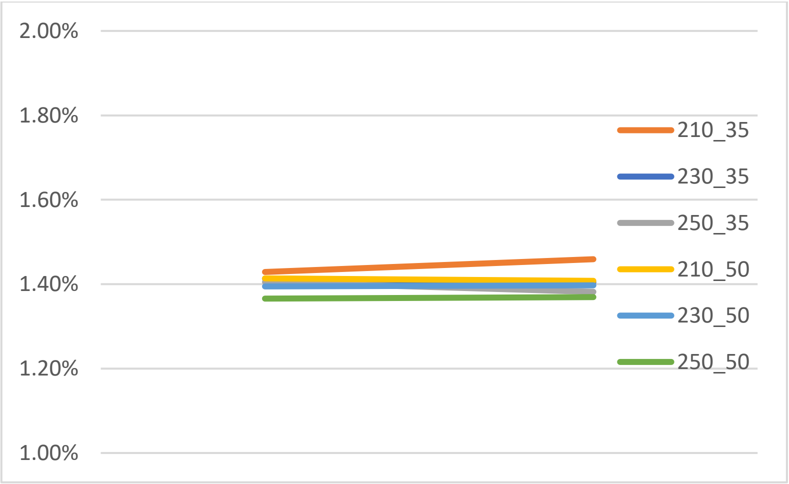

Figure S3. Shrinkage rate profile at different melt and mold temperatures at a specific volume of 1.1308 (thickness 1.5 mm).

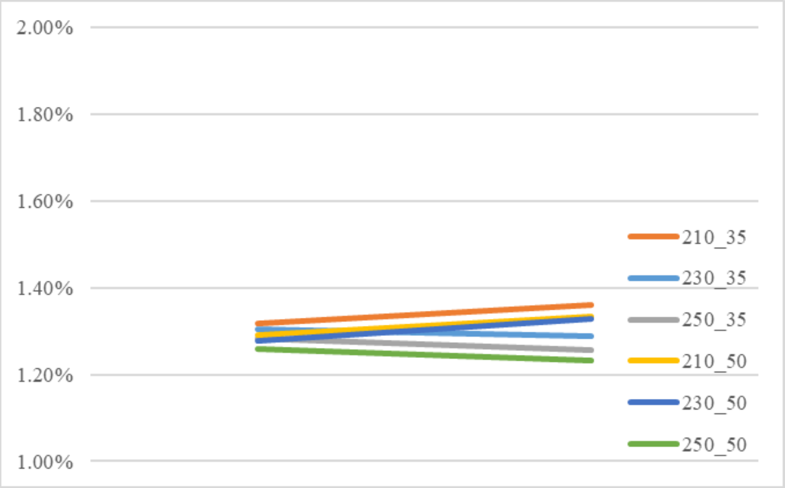

Figure S4. Shrinkage rate profile at different melt and mold temperatures at a specific volume of 1.1267 (thickness 1.5 mm).

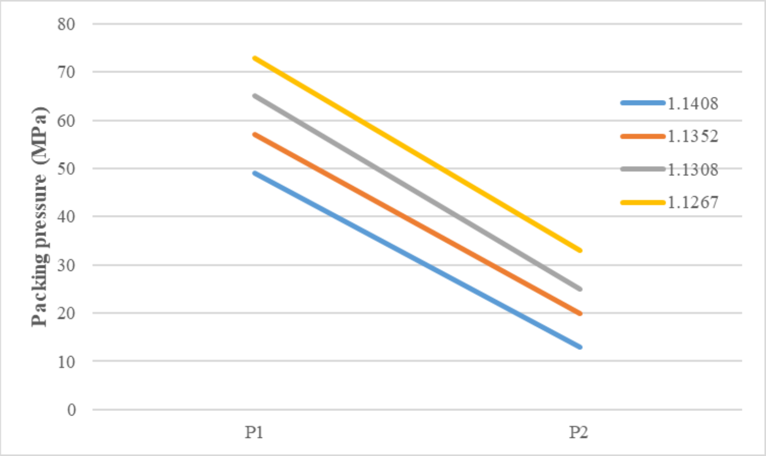

Figure S5. Pressure profile at a melt temperature of 210 °C and a mold temperature of 35 °C (thickness 2 mm).

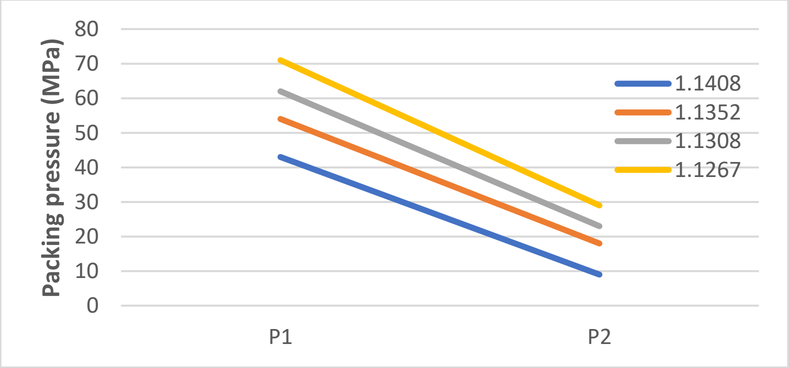

Figure S6. Pressure profile at a melt temperature of 230 °C and a mold temperature of 35 °C (thickness 2 mm).

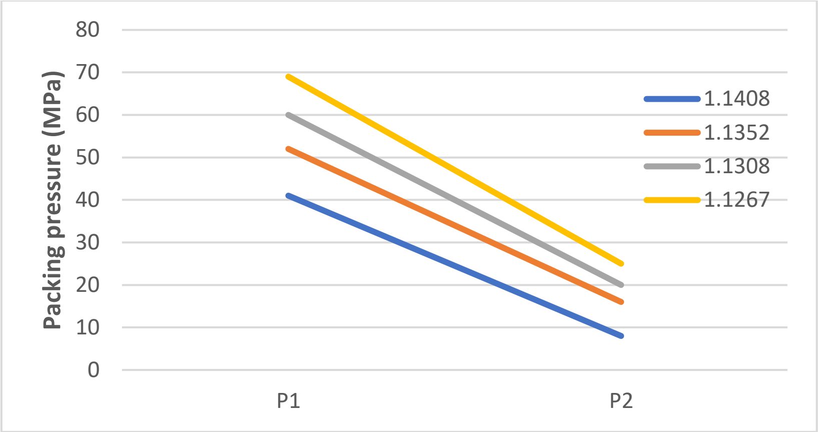

Figure S7. Packing pressure profile at a melt temperature of 250 °C and a mold temperature of 35 °C (thickness 2 mm).

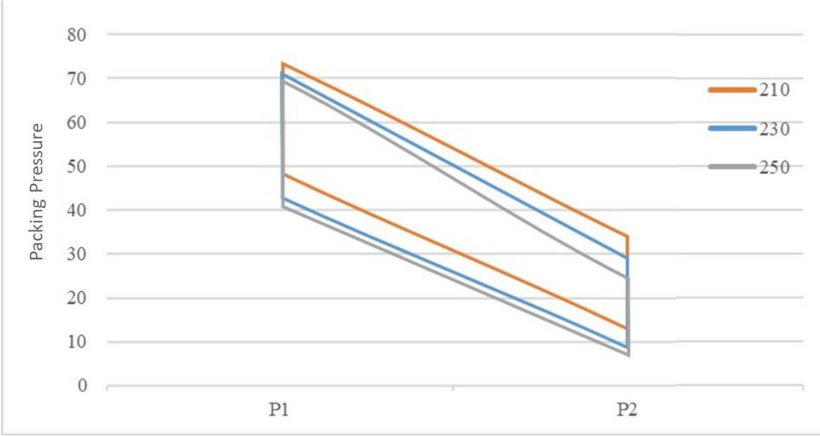

Figure S8. Packing pressure profile at melt temperatures of 210, 230, and 250 °C (thickness 2 mm).

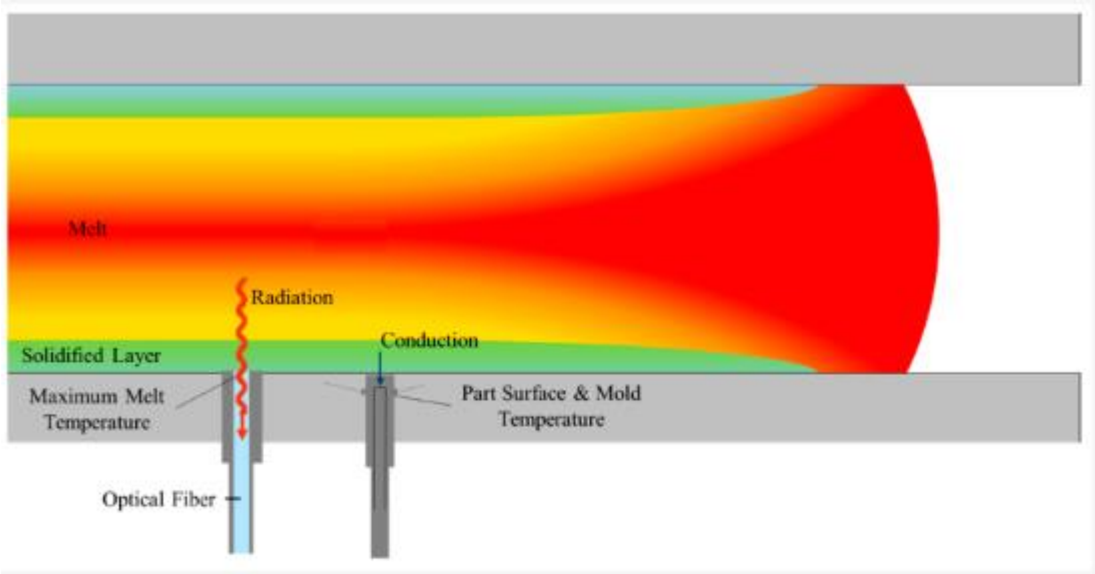

Figure S9. Schematic comparison of temperature measurement methods using a Futaba infrared temperature sensor and a conventional thermocouple.

Table S1. Packing pressure and packing time setting at a thickness of 1.5 mm.

Mold T. (°C)	Melt T. (°C)	Target S. V. (cm^3/g)	Packing pressure (MPa)		Packing time (s)	
			P1	P2	t1	t2
35	210	1.1408	56	11	3.3	2.5
		1.1352	68	18		
		1.1308	76	29		
		1.1267	83	35		
	230	1.1408	53	9	3.7	2.5
		1.1352	65	18		
		1.1308	74	25		
		1.1267	81	34		
	250	1.1408	49	6	3.8	2.9
		1.1352	61	17		
		1.1308	70	24		
		1.1267	79	31		
50	210	1.1408	66	13	3.8	3
		1.1352	79	24		
		1.1308	94	28		
		1.1267	103	37		
	230	1.1408	63	11	4	3.1
		1.1352	76	20		
		1.1308	87	26		

		1.1267	97	33		
	250	1.1408	61	10	4.5	3.3
		1.1352	73	18		
		1.1308	83	24		
		1.1267	94	31		

Table S2. Specific volume at a thickness of 1.5 mm.

Mold T. (℃)	Melt T. (℃)	Target S. V. (cm^3/g)	Real S. V (cm^3/g)		Error (cm^3/g)	
			NG	MID	NG	MID
35	210	1.1408	1.1408	1.1412	0.0000	-0.0004
		1.1352	1.1356	1.1349	-0.0004	0.0003
		1.1308	1.1309	1.1310	-0.0001	-0.0002
		1.1267	1.1270	1.1270	-0.0003	-0.0003
	230	1.1408	1.1410	1.1407	-0.0002	0.0001
		1.1352	1.1353	1.1351	-0.0001	0.0001
		1.1308	1.1308	1.1307	0.0000	0.0001
		1.1267	1.1269	1.1266	-0.0002	0.0001
	250	1.1408	1.1405	1.1409	0.0003	-0.0001
		1.1352	1.1353	1.1354	-0.0001	-0.0002
		1.1308	1.1306	1.1307	0.0002	0.0001
		1.1267	1.1264	1.1265	0.0003	0.0002
50	210	1.1408	1.1405	1.1405	0.0003	0.0003

		1.1352	1.1350	1.1354	0.0002	-0.0002
		1.1308	1.1306	1.1310	0.0002	-0.0002
		1.1267	1.1267	1.1269	0.0000	-0.0002
	230	1.1408	1.1405	1.1408	0.0003	0.0000
		1.1352	1.1349	1.1348	0.0003	0.0004
		1.1308	1.1311	1.1310	-0.0003	-0.0002
		1.1267	1.1267	1.1268	0.0000	-0.0001
	250	1.1408	1.1406	1.1406	0.0002	0.0002
		1.1352	1.1353	1.1351	-0.0001	0.0001
		1.1308	1.1310	1.1309	-0.0002	-0.0001
		1.1267	1.1271	1.1268	-0.0004	-0.0001

Table S3. Shrinkage rate at a thickness of 1.5 mm.

Mold T. (℃)	Melt T. (℃)	Target S. V. (cm3/g)	Shrinkage rate (%)		Deviation (%)
			NG	MID	
35	210	1.1408	1.63	1.68	-0.05
		1.1352	1.55	1.57	-0.02
		1.1308	1.43	1.46	-0.03
		1.1267	1.32	1.36	-0.04
	230	1.1408	1.61	1.60	0.01
		1.1352	1.52	1.56	-0.04
		1.1308	1.41	1.40	0.01
		1.1267	1.30	1.29	0.01

	250	1.1408	1.59	1.61	-0.02
		1.1352	1.50	1.51	-0.01
		1.1308	1.40	1.38	0.02
		1.1267	1.28	1.26	0.03
50	210	1.1408	1.61	1.63	-0.02
		1.1352	1.51	1.50	0.01
		1.1308	1.41	1.41	0.01
		1.1267	1.29%	1.33%	-0.04%
	230	1.1408	1.59%	1.60%	0.00%
		1.1352	1.50%	1.51%	-0.02%
		1.1308	1.39%	1.40%	0.00%
		1.1267	1.28%	1.33%	-0.05%
	250	1.1408	1.57%	1.57%	0.00%
		1.1352	1.48%	1.48%	0.00%
		1.1308	1.37%	1.37%	0.00%
		1.1267	1.26%	1.23%	0.03%

Table S4. Corresponding shrinkage rate at different target specific volumes.

Mold T. (°C)	Melt T. (°C)	Target specific volume (cm^3/g)			
		1.1408	1.1352	1.1304	1.1267
35	210	1.66 %	1.56 %	1.44 %	1.34 %
	230	1.60 %	1.54 %	1.41 %	1.30 %
	250	1.60 %	1.51 %	1.39 %	1.27 %

	210	1.62 %	1.51 %	1.41 %	1.31 %
50	230	1.59 %	1.50 %	1.40 %	1.30 %
	250	1.57%	1.48%	1.37%	1.25%

Table S5. Packing pressure and packing time setting at a thickness of 2 mm.

Mold T. (°C)	Melt T. (°C)	Specific S. V. (cm^3/g)	Packing P. (MPa)		Packing time (s)	
			P1	P2	t1	t2
35	210	1.1408	49	13	6.3	3
		1.1352	57	20		
		1.1308	65	25		
		1.1267	73	33		
	230	1.1408	43	9	6.7	3.9
		1.1352	54	18		
		1.1308	62	23		
		1.1267	71	29		
	250	1.1408	41	8	7.2	4.5
		1.1352	52	16		
		1.1308	60	20		
		1.1267	69	25		
50	210	1.1408	58	11	7.2	3.6
		1.1352	75	22		
		1.1308	86	26		
		1.1267	95	33		

230	1.1408	56	9	7.5	4.4
	1.1352	69	19		
	1.1308	81	24		
	1.1267	90	30		
250	1.1408	52	8	8.3	6
	1.1352	65	17		
	1.1308	75	23		
	1.1267	86	28		

Table S6. Specific volume at a thickness of 2 mm.

Mold T. (°C)	Melt T. (°C)	Target S. V. (cm^3/g)	S. P. (cm^3/g)		Error (cm^3/g)	
			NG	MID	NG	MID
35	210	1.1408	1.1408	1.1408	0.0000	0.0000
		1.1352	1.1352	1.1351	0.0000	0.0001
		1.1308	1.1310	1.1310	-0.0002	-0.0002
		1.1267	1.1268	1.1269	-0.0001	-0.0002
	230	1.1408	1.1408	1.1407	0.0000	0.0001
		1.1352	1.1352	1.1351	0.0000	0.0001
		1.1308	1.1311	1.1308	-0.0003	0.0000
		1.1267	1.1269	1.1268	-0.0002	-0.0001
	250	1.1408	1.1407	1.1408	0.0001	0.0000
		1.1352	1.1350	1.1353	0.0002	-0.0001
		1.1308	1.1310	1.1309	-0.0002	-0.0001

		1.1267	1.1265	1.1266	0.0002	0.0001
50	210	1.1408	1.1407	1.1408	0.0001	0.0000
		1.1352	1.1350	1.1353	0.0002	-0.0001
		1.1308	1.1308	1.1306	0.0000	0.0002
		1.1267	1.1270	1.1268	-0.0003	-0.0001
	230	1.1408	1.1411	1.1410	-0.0003	-0.0002
		1.1352	1.1351	1.1354	0.0001	-0.0002
		1.1308	1.1307	1.1310	0.0001	-0.0002
		1.1267	1.1266	1.1266	0.0001	0.0001
	250	1.1408	1.1410	1.1410	-0.0002	-0.0002
		1.1352	1.1350	1.1351	0.0002	0.0001
		1.1308	1.1310	1.1308	-0.0002	0.0000
		1.1267	1.1264	1.1268	0.0003	-0.0001

Table S7. Real shrinkage rate at a thickness of 2 mm.

Mold T. (°C)	Melt T. (°C)	Target S. V. (cm^3/g)	Shrinkage rate (%)		Deviation
			NG	MID	
35	210	1.1408	1.49	1.51	-0.02
		1.1352	1.37	1.39	-0.02
		1.1308	1.29	1.28	0.01
		1.1267	1.19	1.19	0.01
	230	1.1408	1.44	1.45	-0.01
		1.1352	1.36	1.38	-0.03

		1.1308	1.28%	1.26%	0.02
		1.1267	1.18	1.17	0.00
	250	1.1408	1.43	1.44	-0.01
		1.1352	1.36	1.33	0.03
		1.1308	1.25	1.24	0.01
		1.1267	1.16	1.17	-0.01
50	210	1.1408	1.47	1.50	-0.03
		1.1352	1.35	1.34	0.01
		1.1308	1.30	1.31	-0.01
		1.1267	1.14	1.16	-0.03
	230	1.1408	1.43	1.42	0.01
		1.1352	1.34	1.36	-0.02
		1.1308	1.28	1.29	-0.01
		1.1267	1.13	1.13	0
	250	1.1408	1.40	1.41	-0.01
		1.1352	1.32	1.35	-0.03
		1.1308	1.23	1.21	0.02
		1.1267	1.13	1.11	0.02
